# Antimicrobial Resistance in Rural Settings in Latin America: A Scoping Review with a One Health Lens

**DOI:** 10.3390/ijerph18189837

**Published:** 2021-09-18

**Authors:** Maria Luisa Medina-Pizzali, Stella M. Hartinger, Gabriela Salmon-Mulanovich, Anika Larson, Maribel Riveros, Daniel Mäusezahl

**Affiliations:** 1 School of Public Health and Administration, Universidad Peruana Cayetano Heredia, Av. Honorio Delgado 430, San Martin de Porres, Lima 31, Peru; maria.medina.p@upch.pe (M.L.M.-P.); gsalmonm@pucp.edu.pe (G.S.-M.); larsona@uw.edu (A.L.); 2 Department of Epidemiology and Public Health, Swiss Tropical and Public Health Institute, Socinstrasse 57, 4057 Basel, Switzerland; daniel.maeusezahl@unibas.ch; 3 Swiss Tropical and Public Health Institute, University of Basel, Petersplatz 1, 4051 Basel, Switzerland; 4 Institute for Earth, Nature and Energy at Pontificia Universidad Catolica del Peru, Av. Universitaria 1801, San Miguel, Lima 32, Peru; 5 Department of Environmental and Occupational Health Sciences, University of Washington, Seattle, WA 98195, USA; 6 School of Medicine, Universidad Peruana Cayetano Heredia, Av. Honorio Delgado 430, San Martin de Porres, Lima 31, Peru; maribel.riveros@upch.pe

**Keywords:** anthropogenic activities, livestock, environment, Latin America, one health, antimicrobial resistance

## Abstract

Antimicrobial resistance (AMR) in rural Latin America is not fully understood. The transmission pathways are partially known since research predominantly focuses on the urban hospital setting. The contribution to AMR from environmental factors is usually only mentioned in large-scale animal production. To understand the state of the literature on AMR in rural LA, we carried out a scoping review using the One Health (OH) perspective. OH recognises the concomitant contributions and interconnectedness of humans, animal, and the environment, thus, we used the OH perspective to select those articles adopting a holistic view of the problem. We searched original articles in English, Spanish, and Portuguese in four peer-reviewed databases and included 21 publications in the analysis. We charted data on bibliometrics, design, data collection sources, and instruments. We identified the human, animal, and environmental contributions to AMR in rural locations, and information gaps on AMR transmission routes and AMR drivers. Intensive and non-intensive animal production systems and agricultural practices were the most frequently found human contributions to AMR. Poultry, swine, cattle, and fish were the most frequent livestock mentioned as sources of AMR bacteria. Animal carriage and/or transfer of AMR determinants or bacteria was recognised as the primary contribution of livestock to the problem, while water, soil, and farming were predominant environmental contributions. We found that only 1 article out of 21 considered the OH approach as a framework for their sampling scheme, whereas 5 out 21 discussed all the three OH components. There were hardly any descriptions of humans or human waste as reservoirs for AMR in rural locations, and rural health centres or hospitals and wildlife were not represented. No studies identified mining as an anthropogenic activity driving AMR. More OH-oriented studies, with emphasis on molecular approaches—for identification and comparison of AMR genes—are sorely needed to understand better the existence of a network of interconnected transmission routes in rural Latin America and provide efficient strategies to prevent further AMR emergence.

## 1. Introduction

Antimicrobial resistance (AMR) is a global public health issue [1,2,3], which is occurring among a wide range of microorganisms with increasing prevalence [2]. AMR is a natural phenomenon [1] that can arise from mutations or through AMR genes transmitted within the same microbial species or horizontally among different species [4,5]. Drug-resistant microorganisms are found in people, animals, food, plants, and the environment and threaten our ability to treat common infections [6]. The One Health (OH) concept is used by the United Nations (World Health Organization, World Organization for Animal Health, and Food and Agriculture Organization) to address global health issues, specifically AMR [7]. The OH concept recognises that “human health and animal health are interdependent and bound to the health of the ecosystems in which they exist” [8]. Several national actors adopt a OH perspective when analysing human, animal, and environmental health [9] or apply a OH approach when implementing control measures [10].

Using the OH lens, we find that in humans, AMR is linked to diverse social factors such as non-adherence to regimens or doses, self-medication, and misperceptions regarding antibiotics [11]. Likewise, diverse anthropogenic activities also drive AMR, among them, the presence of hospitals, industries (i.e., mining, pharmaceutical), and urbanization, generating chemical waste and faecally-contaminated water. These activities pollute the environment with antimicrobials, biocides, heavy metals, bacteria with AMR, and AMR genes which are known drivers of AMR [3,12]. On the other hand, wild or domestic animals, animal production, and animal-based agricultural systems are linked to the spread of AMR to humans and the environment [13]. The excessive or inadequate use of antibiotics in animal farming drives AMR [14] by favouring the increase in the number of resistant strains in farm animals, animal origin-food, and animal manure [15,16]. AMR bacteria in animal-origin food may cause foodborne-disease outbreaks, remain as commensals, and bring about drug-resistant infections later [17]. Aquaculture stands out as a very important AMR contributor because it promotes significant genetic exchange and recombination at a fast rate [18]. In addition, antimicrobial residues in feed and animal waste pollute soil and water [14]. Thus, animal-based agricultural systems and fish farming are a direct source for the transmission of AMR bacteria and antimicrobial residues to wildlife, humans, and the whole ecosystem [14,19]. In short, the most important routes for the spread of AMR drivers into the environment are communal and industrial wastewater, human and animal waste (i.e., the application of animal manure and sewage sludge for land fertilising), and aquaculture [12].

Specifically, in Latin America (LA), human and veterinary use of antibiotics is loosely regulated, and antibiotics are readily available over the counter without prescription [20,21,22]. In addition to urban clinical settings [23,24,25], rural environments have been linked to AMR in Latin America [17,26,27]. In such settings, small-scale animal farming is predominant [28,29] and animal excreta are a key driver of faecal contamination in domestic human environs [30]. Thus, rural settings may be an important eco-sphere for the dissemination of AMR. However, in rural settings of low- and middle-income countries—including Latin American countries [22], AMR transmission routes are not clearly documented, and the evidence shown is limited. Reasons may include: AMR surveillance of agricultural and animal production management is limited and/or poor [14,22]; the available literature is sparse and often lacks scientific rigor, providing insufficient information for an adequate overview [13,22], and there is scarcity of OH research, involving the human, animal, and environment domains [13].

This study aims at identifying research that focuses on AMR in rural settings in Latin America (LA) using the OH lens as a framework to identify their human, animal, and environmental contributions to AMR. In addition, this work seeks to pinpoint knowledge gaps on AMR transmission routes and AMR drivers to inform researchers and raise awareness in policymakers. Specifically, this review answers the following research question: What are the concomitant contributions of humans, farm animals, and the environment on antimicrobial resistance in rural settings in LA?

## 2. Materials and Methods

The steps included for our review followed the checklist outlined in the PRISMA Extension for Scoping Reviews (PRISMA-ScR) by Tricco et al. [31] and the guidelines provided by Peters et al. [32].

### 2.1. Inclusion Criteria

The study period spanned from January 2001 until December 2018. Only original, peer-reviewed articles published in Spanish, Portuguese, and English were considered. A search strategy was designed using terminology associated with the three domains of OH and antimicrobial resistance in rural geographic environments for human populations in Latin American countries. We developed and refined key search terms with online databases prior to the article search (Table A1).

### 2.2. Exclusion Criteria

We excluded grey literature. Reviews and meta-analyses were excluded, but their reference lists were scanned. Articles not acknowledging or discussing all three OH components were excluded.

We consulted the following electronic databases concentrating on peer-reviewed articles only: PubMed (biomedical sciences), Web of Science (multidisciplinary), Scopus (multidisciplinary), and SciELO (multidisciplinary for LA and the Caribbean). We carried out the search from 13 November to 3 December 2018. All articles were uploaded to a Mendeley database [33].

**Figure 1:**
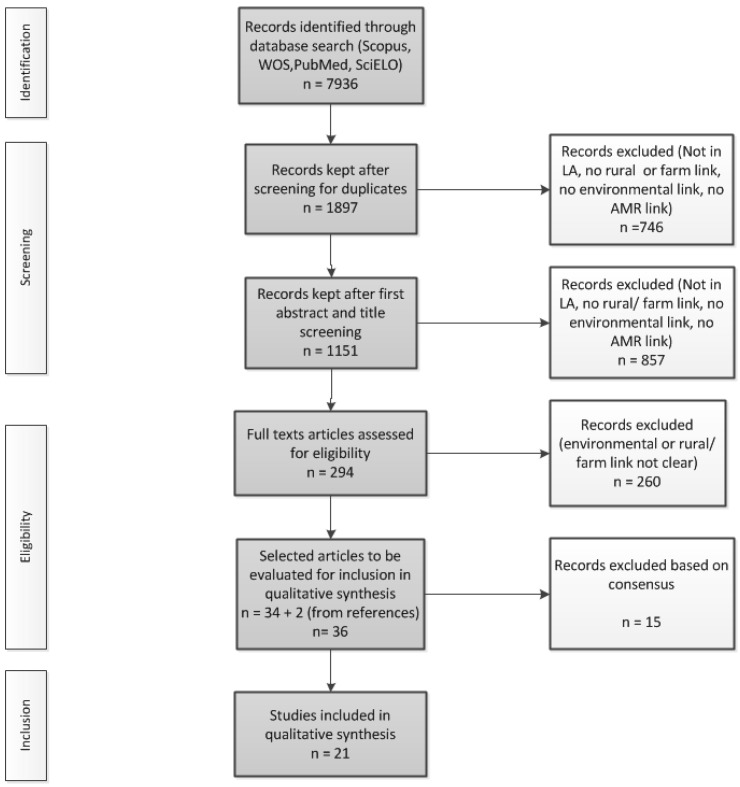
Flowchart of the study selection process.

A multi-step process was applied for the analysis of inclusion (Figure 1). Our initial search produced 7936 articles. After screening for duplicates and evaluating the search criteria (AMR link, rural link, environmental link, animal or agriculture link, and LA country link), we screened 1151 publications. After evaluating the titles and abstracts of the articles in our Mendeley database, we created an initial list of 294 articles meeting the eligibility criteria. These selected articles were then read in full and evaluated for inclusion; 21 publications were finally included for the qualitative synthesis. Five reviewers conducted all stages of the scoping review, from relevance screening to data extraction. The five reviewers individually selected the studies for each phase of elimination. In the process, the reviewers met and discussed each study they had identified, and jointly agreed on including or excluding the study for analysis. If no agreement could be reached, the senior author (SH) decided.

### 2.3. Article Selection

We defined relevant publications as any original peer-reviewed article published between 1 January 2001 to 3 December 2018; in English, Spanish, and Portuguese language that presented data from LA countries only (Argentina, Bolivia, Brazil, Chile, Colombia, Costa Rica, Cuba, Dominic Republic, Ecuador, El Salvador, Guatemala, Haiti, Honduras, Mexico, Nicaragua, Panama, Paraguay, Peru, Uruguay, and Venezuela); referred to a rural human setting and to agricultural and/or animal-based food production activities, linked to environmental aspects, and focused on the topic of AMR. To be included, an article needed to refer to any aspects of AMR and to partially or totally match the concept of AMR as defined by the WHO: “Antimicrobial resistance (AMR) is the ability of a microorganism (like bacteria, viruses, and some parasites) to stop an antimicrobial (such as antibiotics, antivirals, and antimalarials) from working against it. As a result, standard treatments become ineffective; infections persist and may spread to others” [34].

Considering an OH perspective, and using it as a screening tool, we searched for articles that linked to human populations in rural settings (e.g., rural hospitals or health services, rural communities, farms), had a connection with agricultural and/or animal production activities (e.g., cattle, fish, poultry, swine farming or animal keeping, transportation, slaughtering, meat-processing), and described a strong connection with AMR and the environment. All the inclusion criteria needed to be met, and thus, the criteria needed to be explicitly stated and/or discussed in the article. Finally, we excluded studies on AMR that focused only on topics that were remotely linked to agricultural and animal-based-food production activities, such as articles describing urban hospital settings or industrial food production settings; peripherally related to environmental aspects such as publications dealing with economic impacts; loosely linked to AMR discussing molecular/kinetics analyses of enzymes responsible for AMR, and describing research in parks and zoos within urban spaces, since they would not qualify as “rural”.

### 2.4. Data Management and Characterisation/Charting

We tabulated data extracted from the selected articles, including authors, year of publication, title, research objectives, DOI, URL, location of the study, language, and summary of the findings. We used a charting spreadsheet established a priori as a guide, which was established through team discussions when reviewing the preliminary results. If investigators from individual studies were contacted, their clarifications were included.

### 2.5. Analysing, Summarising, and Reporting the Results

The analysis and synthesis of literature included quantitative analysis (i.e., descriptive statistics) and qualitative analysis (i.e., content analysis). For the qualitative analysis, reviewers extracted common themes that emerged from the findings, and the team discussed the results. Each article was analysed to identify the approach to study AMR and findings regarding each theme.

## 3. Results

### 3.1. Literature Profile

A total of 19 articles were included in the analysis, and 2 articles were added after browsing their references (Figure 1).

The 21 studies included in the analysis originated from 8 Latin American countries: Brazil, Ecuador, Colombia, Argentina, Chile, Mexico, El Salvador, and Peru, with Brazil providing the most articles (9 of 21). All articles but one were published in 2010 and onwards, peaking with five publications in 2014. All of them were written in the English language. Nineteen studies were funded by research funders, public agencies; two did not declare their funding source. One study was partially funded by a microbiological laboratory which supplied *Salmonella* spp. strains, and three studies were partially funded by private LA universities.

All studies were quantitative and had a cross-sectional design except for one, which was qualitative. Two articles addressed AMR only through molecular methods, six combined phenotypic profiling and molecular techniques (PCR), and eight only through microbiological methods. Three studies analysed microbiological and epidemiological data. Only one article included a chemical identification analysis of antibiotics in samples in addition to molecular genetic analysis. All eligible research works addressed AMR in a rural site, but four studies also took samples in urban or peri-urban sites for comparison. In one study, we assumed the location was rural (poultry production sites), based on current poultry production practices, but it was not explicit in the article. Table 1 presents the 21 publications included in our review and their characteristics are summarised in Table 2. Table 3 outlines the methods used in each of the research works.

### 3.2. Antimicrobial Resistance through the One Health Lens

**Figure 2:**
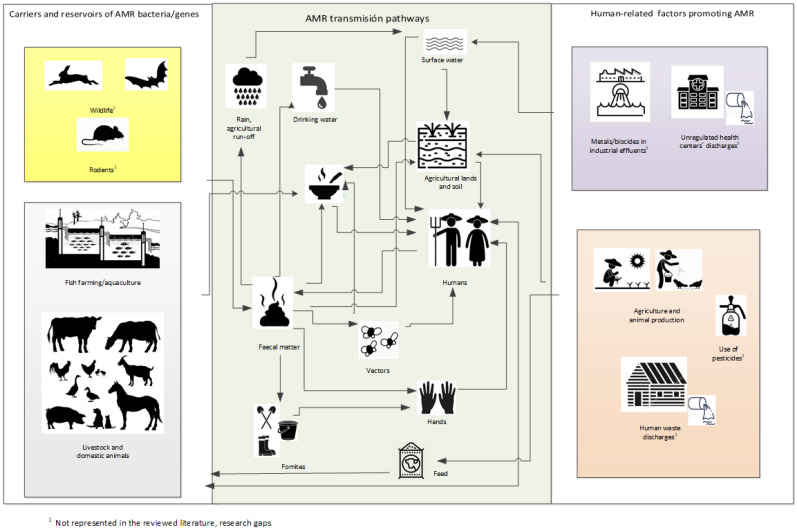
Proposed pathways for the spread of antimicrobials and/or AMR genes in rural settings, based on the publications included in the review.

Figure 2 shows AMR contributions from animal, environmental, and human domains, applying the OH perspective; it describes how various specific human activities, animal-related factors, and environmental factors are connected based on the information extracted from the selected articles.

As shown in Table 1, in most articles, only two OH components were considered, either in the discussion or the description of the sampling procedures. Only Braykov et al. [37], Brisola et al. [38], dos Vieira et al. [47], Miranda et al. [48], and Palhares et al. [49] discussed all three components, but their study designs did not include sampling for all of them. Pehrsson et al. [50] took samples of human, animal, and environmental origin but did not discuss the relevance or impact of their results on animal health.

#### 3.2.1. Human Contribution

Anthropogenic drivers of AMR included intensive and non-intensive (small-scale/extensive) animal production systems [35,36,37,38,39,40,41,42,43,44,45,46,47,48,49,50,51] and agricultural practices [49,50,52,53,54], such as the use of recycled or composted animal or human manure as fertiliser. Resende et al. [53] showed microbiological evidence of survival of AMR bacteria after biodigestion treatment of cattle manure, which could contaminate soils when applied as fertiliser. Camotti et al. [52] found that each type of manure used as fertiliser in agricultural soils had a unique concentration of antibiotic residues and AMR genes, specific to the particular animal production system it originated from. Perhsson et al. [50] and Kalter et al. [42] identified inadequate human excreta management as an important human AMR-promoting factor in rural sites. Only three articles [35,42,50] identified unrestricted, unregulated, or recent use of antimicrobials in humans as a human contribution to the problem. Cicuta et al. [55] did not identify any human input but acknowledged the need to have an interdisciplinary approach to implement human and animal health-oriented research. Only Perhsson et al. [50] gave direct evidence of the human role in the generation of AMR; they proved that humans modify microbiomes and resistomes in rural settings by interacting with animals and the environment by means of horizontal transfer of AMR determinants.

#### 3.2.2. AMR and Contributions from Animals

Poultry was the most common livestock [35,37,39,42,43,44,45,49,50,52], but fish [36,48], swine [38,40,41,42,43,49], and cattle [41,42,46,49,51] were also studied. Only dos Vieira et al. [47] focused on AMR in shrimp production. Some studies considered the input of other domestic animal species [41,42,43,55] such as sheep, ducks, pigeons, horses, dogs, or guinea pigs. Corzo-Ariyama et al. [54] did not specify the type of animal under study; their focus was solely on identifying AMR patterns from bacteria in produce; therefore, they recognised the animal role in the spread of AMR more generally. By far, animal AMR carriage and/or transfer of AMR determinants or bacteria was the most widely identified animal contribution [36,37,38,44,46,47,48,50,51,52,53,54], while several studies mentioned the inadequate or unregulated use of veterinary antimicrobials [39,40,41,43,47,48,49]. The role of food of animal origin in the spread of AMR bacteria in the human food chain was mentioned as well [35,36,38,39,42,46,47].

In contrast, Cicuta et al. [55] did not acknowledge any of the above-mentioned contributions of animals to the generation and spread of AMR, but only discussed the phenotypic resistance screening results and their likely cellular resistance mechanisms.

Armas-Freire et al. [35], Brisola et al. [38], Campioni et al. [39], Mattiello et al. [44], and Lopez et al. [51] showed strong evidence of the animal role as reservoirs or carriers of AMR genes obtained by molecular methods—allowing identification and comparison of AMR genes [56]—combined with phenotypic resistance testing—based on viable culturable bacteria [57]. Santamaria et al. [46] and Pehrsson et al. [50] used only molecular methods to study AMR, but the latter applied metagenomics to compare entire resistomes. Camotti et al. [52] used a combined molecular and chemical methodology to identify AMR genes and antimicrobial molecules. Braykov et al. [37], Kalter et al. [42], and Rodriguez et al. [45] included an epidemiological methodology and one of them provided sound evidence regarding the risk and protective factors for AMR presence in humans. One of the main risk factors for AMR were children’s or household members’ recent antibiotic use. At the same time, AMR was less often described among older children and those living in a community where a greater proportion of homes consumed home-raised chicken [42]. Most of the above-mentioned articles proposed a one-way transmission pathway of AMR genes from animals to the environment. However, Brisola et al. [38] and Pehrsson et al. [50] proposed a more complex scenario of interactions where the dissemination of AMR occurs simultaneously and in two opposite directions linking all reservoirs: human, animal, and environmental, although Brisola et al. [38] pointed at the animal reservoir as the origin.

#### 3.2.3. Environment Contribution

More than half of the articles identified water as a contributing factor for AMR spread [36,37,38,41,42,46,47,48,49,50,51,52,54]. Other environmental inputs included: soil [37,38,46,49,50,51,52,53,54], farm/bird coop’s environment (which included surfaces, feed, shoe soles, and/or hands of workers) [37,44,45,54], vectors (flies) [40], and pond sediments [47]. Lopez et al. [51] recognised the importance of soil-containing faeces in the contamination of underground and surface water. Santamaría et al. [46] and Palhares et al. [49] highlighted the importance of runoff in disseminating AMR genes into the environment. Lowenstein et al. [43] considered questions about the use of shared animal-human drinking water sources and shared living spaces. Interestingly, Miranda et al. [48] pointed at feed and influent water—as opposed to effluent water—as reservoirs for AMR bacteria in salmon farms. On the other hand, Armas Freire et al. [35] and Cicuta et al. [55] did not address any environmental contributions to the AMR problem.

Brisola et al. [38], Campioni et al. [39], Mattielo et al. [44], Santamaria et al. [46], Pehrsson et al. [50], Lopez et al. [51], and Camotti et al. [52] produced sound evidence regarding the role of the environment in the maintenance and dissemination of AMR. All these articles agreed that the faecally-contaminated environments are a persistent source or reservoir for AMR bacteria from which AMR could easily disseminate. Most of them considered animals as the contamination source but Pehrsson et al. [50] verified the contribution of both animal and human faecal matter in this contamination of the environment. Strong microbiological/epidemiological evidence was provided by Braykov et al. [37] and Kalter et al. [42] for the role of the environment in the spread of AMR.

### 3.3. Information Gaps

As shown in Figure 2, neither of the selected articles investigated nor identified the contribution of effluents of rural hospitals or health services to the environment and their impact on rural populations, animals, and ecosystems. Four articles included human faecal samples in their studies, but human waste collective discharges were not sampled. Likewise, the link between mining and AMR in rural settings was not the focus of any of the eligible studies, despite the role of metals as drivers of AMR [12] and the contribution of mining to metal pollution [58,59]. Moreover, wild animal reservoirs and/or their contribution to the AMR problem in rural locations were not discussed in the selected articles. Even though many studies published in Portuguese or Spanish language were found at the initial steps of the search, none of them met the inclusion criteria, so they were not represented in our selection.

## 4. Discussion

This review identified key contributors to AMR in LA considering the OH concept. The following anthropogenic activities were identified as drivers for AMR dissemination in rural Latin American settings: animal husbandry, fish farming, agriculture, and other related practices such as animal waste recycling. The carriage and/or transfer of AMR determinants were the most frequent animal contributions, in addition to the inadequate or unregulated use of veterinary antimicrobials and the role of food of animal origin in the spread of AMR bacteria in the human food chain. Water was the most commonly identified environmental contributor for AMR spread but also soil, farm/bird coops, vectors (flies), and pond sediments were also important contributors mentioned.

Nearly half of the eligible studies showed robust evidence confirming the human, animal, or environmental contributions to the generation or the spread of AMR. However, only one study [50] provided evidence embracing a OH framework to suggest a global scenario in which all the reservoirs—human, animal, and environmental—contribute to the problem, sharing AMR genes through horizontal transfer. Rather than illustrating a mere pathway, this work embraces the numerous interactions between human, animal, and environmental domains portraying an intricate network of AMR spread. Pehrsson et al. [50] took samples of human, animal, and environmental origin simultaneously and compared their resistomes, allowing them to produce strong evidence for the interconnectedness of human, animal, and environmental drivers of AMR. As these AMR drivers converge, the environment might function as both a reservoir and a bridge for antimicrobial determinants giving rise to other potential pathways of AMR transmission to non-contaminated wildlife, humans, and animals [12].

Most of the assessed articles studied the AMR problem from a single viewpoint or emphasised one of the OH components. Five studies [37,38,47,48,49] considered the importance of animal, human, and environment inputs to the AMR generation and spread, but did not collect samples from all these interconnected sources or omitted discussing the results in an integrated manner. The reasons were not explicit.

One of the most important insights from our study is the scarce research on humans or human waste as sources of AMR determinants in rural locations described in the Latin American literature. Studies focusing on AMR in rural health centres or rural hospitals were not found in the articles eligible for analysis. However, the impacts of rural hospital effluents on the environment and hence, on human and animal microbiomes cannot be ignored [60], mainly because wastewater collection and treatment in rural settings are significantly reduced or absent compared to urban settings in LA [61,62].

We found some information gaps in the selected literature. Mining is an important economic activity in many countries in LA [63] and it has been identified as a contributor to the spread of AMR, however, we did not find any studies on the topic. Mining activities lead to the release of metal-containing effluents into the environment, driving AMR in bacteria due to shared mechanisms of resistance to both metals and antimicrobials [12].

Wildlife is a neglected likely significant contributor to the spread of AMR in rural LA. Although it was not identified as a contributor in the eligible articles, the interaction between humans, wild animals, and farm and domestic animals occurs in rural settings, sharing AMR bacteria [64]. Thus, studying wild animals’ AMR gene sources and their genetic similarity in farm animals, human, and environmental reservoirs in LA, should be the focus for future research worth working on. More public health research focusing on wildlife is needed to better understand the impacts of human activities on the environment (habitat fragmentation, land-use change, urbanisation) and the role of wildlife species—as reservoirs, melting pots, and/or vectors for AMR determinants—in the dissemination of resistance [65]. Our findings underscore the importance of adopting an OH approach as a framework for the design of future studies aimed at understanding the interconnections among its three components to assess AMR more efficiently and propose better strategies to prevent AMR emergence in LA.

Only one article used qualitative methods [43]. Qualitative approaches are useful when trying to elucidate the reasons behind practices, knowledge, attitudes and perceptions, and prove useful in understanding the complexity of AMR transmission pathways. We suggest incorporating a qualitative approach in AMR research since it could be a significant added value to quantitative studies. Mixed-methods approaches allow researchers to identify any contradictions between the quantitative and qualitative findings [66], and could be valuable for identifying deficiencies in sanitation and biosecurity practices in animal production and agricultural systems, knowledge and attitudes regarding these practices, and the structural and economic limitations contributing to AMR dissemination in LA rural settings.

On the other hand, the most robust evidence for the role of animals, humans, and/or the environment in the spread of AMR originated from studies combining molecular methods, phenotypic resistance screening methods, sound study design and sampling, and an integrated OH perspective. A few studies relied upon a combined phenotypic resistance testing and an epidemiological approach, thus identifying risk factors and protective factors for AMR. However, due to the limitations of culturing in assessing AMR, they could not give insight into the specific AMR determinants associated with the AMR phenotypes [56]. Phenotypic resistance profiling enables cultivation of target bacteria. However, assessing AMR through culture-based methods carries an inherent bias since these methods cannot detect cells in a viable but non-culturable state [57]. In contrast, molecular methods provide information regarding the underlying mechanism of resistance, identifying the determinants for that resistance, even if they are not always expressed in the host bacteria [56,67]. In addition, with the use of genomic tools, typing, comparing, and tracing specific allele profiles, it is now possible [68]. Thus, it is necessary to apply molecular methods along with phenotypic profiling methods to have a more detailed and complete picture when assessing AMR [57]. We believe that studies focusing on the total environment using microbiological, epidemiological, and molecular approaches in an integrative way are needed to better understand the existence of a network of interconnected transmission routes. Additionally, given the cross-sectional nature of the eligible studies, they could not demonstrate the directionality of their proposed pathways of transmission of AMR, which—in most cases—pointed at a one-way path only, from animals to the environment.

It is important to note that only one study in Brazil [47], mentioned that local governmental agencies were concerned with the results of the antibiotics’ indiscriminate use in aquaculture. However, no other mention was made to the uptake of research findings by local authorities or any other local actors in any sector. This finding may imply the need of a more effective dissemination of scientific findings from academia to government agencies and local actors in LA. Likewise, an OH approach to provide robust evidence on AMR emergence and transmission is key to translate AMR’s research results when designing public health and animal production policies.

We did not include grey literature, which we believe would have enriched our findings. There was no systematic way to search for country-level surveillance reports. Since most literature reviews only include publications in the English language, we purposedly looked for articles produced in LA written in Spanish and Portuguese languages, finding a considerable number during the screening process. However, none of them fulfilled the eligible criteria for this review. This limitation may be due to the kind of settings in which these studies have been conducted—urban as opposed to rural—and the AMR perspective applied, which may be one-sided, favouring any of the OH components but not comprising the three domains, as we specified in our inclusion criteria. Since AMR was recognised as a global threat to public health, virtually all countries adopted a national action plan to tackle the problem [69]; however, actions developed in LA may not have had an explicit focus on an integrated OH approach.

Other reasons may explain the scarcity of truly integrated OH research works in LA—as in other low- and middle-income regions. Establishing OH research involves facing barriers such as lack of OH training and expertise, and difficulty in establishing collaboration among multiple and cross-sectoral actors—resulting in scarcity of multidisciplinary training programs—and limited government support and research funding [70,71]. Funding bias could partially explain the absence of articles written in these languages: most comprehensive and well-funded AMR studies adopting an OH approach tended to be published in English.

## 5. Conclusions

This scoping review on AMR in rural settings in LA identified the human, animal, and environmental contributions to AMR using an OH lens, and pinpointed the information gaps on AMR transmission routes and AMR drivers in the literature. Human activities contributed to the spread of AMR through animal husbandry (mainly poultry), fish farming, agriculture, and animal waste recycling (composting). Farm animals contributed by carrying and/or transferring resistant genes or resistant bacteria. Main environmental contributors are faecally-contaminated water, contaminated soil or pond sediments, and farm environments.

Adopting an OH lens proved useful as a framework to determine whether the selected articles considered the impact of AMR on the three aspects of health, animal, human, and environment, and to what extent they did so. However, a small percentage of articles took into account the three OH components in the sampling or in the discussion. Thus, we recommend following the OH approach as a framework for the design of future studies—emphasising on the use of mixed methods and a combination of approaches—molecular-, epidemiological-, and culture-based—to tackle AMR more efficiently and to tailor strategies to prevent AMR emergence in the region, where these efforts are still scant and considerably needed.

Future research efforts should give more attention to the role of mining, wildlife, and rural hospitals’ or health services’ effluents on the emergence and spread of AMR in rural Latin America, given that these aspects were not identified in the selected literature and were considered information gaps.

## Figures and Tables

**Table 1:** Summary of selected publications.

Citation	One Health Component ^1^	Human Contribution to AMR	Animal Contribution to AMR	Environment Contribution to AMR	Location	Important Results (Summary)
Armas-Freire 2015 [35]	AH, HH	FQ ^3^ resistance linked to humans, especially in clinical settings where its use is widespread.	FQ resistance linked to food-producing animals, no restriction to the use of FQ ^3^.	Water samples collected but not discussed.	Ecuador	Higher FQ ^3^ resistance in *E. coli* isolates from chickens than in rural human isolates. The latter showed higher rates of *qnrB* genes compared to chicken isolates. Urban clinical human isolates: low occurrence of *qnrB* genes.
Barbosa 2014 [36]	HH, EH	Expansion of aquaculture/incorrect animal husbandry practices.	Farm animals’ pathogens can colonise fish and become carriers of AMR.	Water gets polluted with animal faeces.	Brazil	*E. coli* strains isolated from fish for human consumption, 43% were EPEC. MDR ^12^ was high in isolates.
Braykov 2016 [37]	HH, AH, EH	Animal production systems boost and are sources for AMR. Broad spectrum antibiotics used in humans contributed to AMR in poultry.	Potential extrinsic sources of resistance: birds could become colonised by resistant strains from hatcheries.	Surfaces of poultry coops: AMR profiles most similar to samples from poultry.	Ecuador	Higher levels of AMR in bacteria from production versus household birds. Prevalence of AMR in production birds declined with bird age.
Brisola 2019 [38]	HH, AH, EH	Pig farming production systems contaminate environment and spread AMR/MDR ^12^ resistant genes.	Pig faeces contaminated environment with *E. coli* carrying MDR genes.	MDR ^12^ isolates found in water and soil.	Brazil	*E. coli* isolates in pig faeces, water, and soil samples: 37.04% showed MDR ^12^, 7.41% were ESBL ^10^ producers. Most MDR ^12^ strains presented a high risk of transmission to humans.
Campioni 2014 [39]	HH, AH	Overuse of QN ^4^ in poultry production spreads AMR.	Resistance to NA ^5^ in *Salmonella enteritidis* isolates from chicken; the pathogen is vehicle for AMR.	Environs not the source of AMR; they become contaminated by chicken breeders sharing the same strains.	Brazil	Some strains isolated from two sources were indistinguishable. Forty-four strains were resistant to NA ^5^. QN ^4^ resistance was present.
Cervelin 2018 [40]	HH, AH	Swine production generates manure and overuses antibiotics, fostering AMR spread through vectors.	Pigs carry AMR zoonotic bacteria, which are pathogenic to animals and humans.	Flies are an environmental factor of importance in the spread of AMR.	Brazil	Resistance detected in 2 out of 4 antibiotics tested (used in human or veterinary medicine). Some farms showed MDR ^12^ bacteria.
Gambero 2018 [41]	HH, EH	Animal farming impacts on quality of surface and groundwater by spreading AMR *E. coli*. Small proportion of *E. coli* resistant to antibiotics used in humans.	Animal faeces contaminate water. High proportion *E. coli* resistant to veterinary antimicrobials.	Water unsafe for human consumption due to *E. coli* concentrations, which foster AMR spread.	Argent-ina	Source of faecal contamination in water is mainly animal residues.
Kalter 2010 [42]	HE, HH	Human use of antibiotics impacts on risk of AMR bacteria carriage in children.	Meat consumption of animals produced commercially drives AMR carriage risk in humans.	Lack of protection of excreta and water play a role in increasing AMR carriage risk in children.	Peru	Individuals taking “any antibiotic” increased children’s risk for resistant *E. coli*. Residence in zones where home-raised chicken was consumed protected against carrying resistant *E. coli*.
Lowen-stein 2016 [43]	AH, HH	Small-scale livestock production could have an impact on the risk of zoonosis and spread of AMR.	Handling and consumption of sick and dead animals: perceived risk factor for AMR spread. Unregulated use of veterinary antimicrobials.	Animal environment sanitation not addressed. Animals and humans sharing water sources and living spaces.	Ecuador	Qualitative study. Handling and consumption of sick and dead animals and over-the-counter purchase of veterinary drugs increase zoonoses risk and AMR spread. Commercial poultry considered less healthy due to antibiotics.
Mattiello 2015 [44]	HH, EH	Use of antibiotics as growth promoters. Improper sanitation favours MDR ^12^ Salmonella.	Animals’ contribution is explained by their carrying AMR pathogenic *Salmonella enterica*.	Poultry house environment is major contributor to AMR. Environmental isolates showed MDR ^12^ to human antibiotics.	Brazil	Poultry house environment produced more AMR isolates. Highest resistance: SA ^6^. Most resistant isolates: *sul* genes. Twenty-one isolates with reduced susceptibility to b-lactams and had *blaTEM*, *blaCMY* and/or *blaCTX-M*.
Rodriguez 2015 [45]	AH, HH	Poultry and egg industry malpractices promote dissemination of pathogenic and resistant *Salmonella* spp.	Hens and eggs carry AMR Salmonella.	Feed and water carried Salmonella. Farm workers’ faecal samples collected but not discussed.	Colom-bia	Salmonella prevalence: 33%; two isolates were MDR ^12^. Farm practices as potential risk factors for Salmonella spread: on-farm feed milling, inappropriate sanitation, egg storage, and inadequate construction material.
Santamaría 2011 [46]	EH, AH	Grassland-based production systems (antibiotics only for disease control) still create reservoirs for AMR bacteria.	Grasslands: cattle are reservoirs of TCN ^7^ resistance genes and are more diverse than environment.	Soil and water are reservoirs of TCN ^7^ resistance genes.	Colom-bia	Remarkable presence of *tet* genes. Predominant distribution of *tet*(W) and *tet* (Q) in both animal and environmental reservoirs. Probable gene transmission from animals to environment.
dos Vieira 2010 [47]	AH, EH, HH	AMR in aquatic environments increased by indiscriminate use of antimicrobials (human and veterinary).	AMR transferred from animals to humans through food.	AMR transferred from shrimps to the environment (pond water and sediment).	Brazil	More than 90.5% of strains of *Escherichia coli* showed a variety of AMR profiles.
Miranda 2002 [48]	AH, HH, EH	Prophylactic therapy in Chilean salmon farming produces higher AMR.	Poor fish farming management and incorrect use of antimicrobials.	Water and feed: likely reservoirs of MDR ^12^ bacteria.	Chile	Gram-negative OXT ^8^-resistant bacteria recovered. MDR ^12^ was frequent.
Palhares 2014 [49]	HH, AH, EH	Antimicrobials in livestock linked to AMR in animals and humans. Inadequate animal husbandry, agricultural, and environmental practices favour presence of Salmonella.	Farm animals, manure, fish farming, and wild animals contribute to AMR Salmonella spread.	Rain, agricultural runoff, and river flow contribute to AMR Salmonella spread.	Brazil	54 different AMR profiles; 49.5% of isolates with AMR. MDR ^12^: 18% of isolates. Link among animal-based agriculture, Salmonella and AMR.
Pehrsson 2016 [50]	HH, EH, AH	Subsistence farming: antibiotic use without prescription and inadequate excreta management favour AMR.	Rural site: backyard farming contributed to AMR spread.	Rural site: soil faecally contaminated with human and animal AMR genes. Limited access to drinking water and sanitation.	Salvador and Peru	Large network of AMR genes shared: microbial communities of humans, animals, and environment.
Lopez ^2^ 2012 [51]	EH, AH	Extensive cattle production impacts environment and animals, creating AMR reservoirs despite low antibiotic use.	TCN ^7^- resistance genes can flow from animal waste to soil and water.	Faecally contaminated soil can pollute underground and surface water.	Colom-bia	No differences in isolates from environmental samples vs. animal samples. TCN ^7^ resistance in grasslands likely caused by horizontal gene transfer from animals to environment.
Camotti 2018 [52]	EH, AH	Use of manure as fertiliser drives accumulation of pharmaceutical residues or induces AMR bacteria in soils.	Poultry, cattle, and swine manure contaminate soils and disseminate AMR bacteria.	Fertilised soils contaminate forest soils.	Brazil	Manure application associated with antibiotic residues and AMR in soils. Swine manure had highest antibiotic concentrations. Extended dairy cow grazing linked to high SA ^6^ resistance.
Resende 2014 [53]	AH, EH	Cattle manure recycling may impact animal, human, and environmental health.	Biodigestion of cattle manure does not guarantee “safe” fertiliser.	Effluent use from ambient temperature biodigesters contaminates soil with AMR bacteria.	Brazil	55.65% of isolated bacteria were MDR ^12^. Some isolates recovered from biodigestor (influent and effluent) were AMR.
Corzo-Ariyama 2019 [54]	HH, EH	Agricultural practices and sewage water contribute to spread of AMR pathogenic *E. coli*.	Compost use, and animal waste: sources of contamination for pathogenic and resistant strains.	Workers’ hands, water, and soil can contaminate produce with pathogenic AMR bacteria.	Mexico	High resistance to TCN ^7^ and AMP ^9^. 3.5% were MDR ^12^. Potential consumer risk: AMR, pathogenicity, and biofilm formation.
Cicuta 2014 [55]	HH, AH	Human contribution not discussed. ESBL ^10^-producing enterobacteria more frequent in humans and animals.	Animals can carry variety of potentially pathogenic ESBL ^10^-producing enterobacteria.	Water samples collected but not discussed nor linked to other samples.	Argent-ina	Neither phenotypically ESBL ^10^ nor CB ^11^-producing bacteria detected.

^1^ HH: Human Health; AH: Animal Health; EH: Environmental Health; ^2^ Both publications belong to the same project and share the same sample; ^3^ Fluoroquinolones; ^4^ Quinolones; ^5^ Nalidixic acid; ^6^ Sulfonamides; ^7^ Tetracyclines; ^8^ Oxytetracycline; ^9^ Ampicillin; ^10^ Extended-spectrum beta-lactamases; ^11^ Carbapenemase; ^12^ Multidrug resistance/multidrug resistant.

**Table 2:** Basic details of included publications.

Study Characteristics	Number (*n* =); Included Articles, *n* (%)	Article Number in References
Type		
Quantitative research	20 (95.2)	[35,36,37,38,39,40,41,42,44,45,46,47,48,49,50,51,52,53,54,55]
Qualitative research	1 (4.8)	[43]
Country of origin		
Brazil	9 (42.9)	[36,38,39,40,44,47,49,52,53]
Ecuador	3 (14.3)	[35,37,43]
Colombia	3 (14.3)	[45,46,51]
Argentina	2 (9.5)	[41,55]
Chile	1 (4.8)	[48]
Mexico	1 (4.8)	[54]
Peru *	2 (9.5)	[42,50]
El Salvador *	1 (4.8)	[50]
Publication year		
2001–2005	1 (4.8)	[48]
2006–2010	2 (9.5)	[42,47]
2011–2016	13 (61.9)	[35,36,37,39,43,44,45,46,49,50,51,53,55]
2017–2019	5 (23.8)	[38,40,41,52,54]
Language		
English	21 (100)	[35,36,37,38,39,40,41,42,43,44,45,46,47,48,49,50,51,52,53,54,55]
Approach used to study AMR		
Microbiological and molecular	6 (28.6)	[35,38,39,44,51,54]
Molecular	2 (9.5)	[46,50]
Microbiological	8 (38.1)	[36,40,41,47,48,49,53,55]
Microbiological and epidemiological	3 (14.3)	[37,42,45]
Chemical and molecular	1 (4.8)	[52]
Other (qualitative)	1 (4.8)	[43]

* The study carried out in El Salvador also included Peru.

**Table 3:** Data collection sources/instruments used in selected publications.

Type of Data Collection	Number (*n* =); Included Articles, *n* (%)	Article Number in References
Animal collection (cloacal swabs, faeces, manure, compost, muscle, eggs, veterinary clinical samples)	16 (76.2)	[35,36,37,38,39,42,44,45,46,47,48,49,50,51,53,55]
Environment collection (soil, water, pond mud, surfaces, workers’ hands, vectors, feed)	18 (85.7)	[35,36,37,38,39,40,41,42,44,45,46,47,48,49,50,51,52,54]
Human collection (faeces)	4 (19.1)	[35,42,45,50]
Questionnaires and interviews, observation	4 (19.1)	[37,42,43,45]
Produce collection	1 (4.8)	[54]

## Data Availability

The data presented in this study are available in Table 1. Additional data are available on request from the corresponding author.

## Appendix A

Table A1 shows the full electronic search strategy used in this review.

**Table A1:** Keywords (with synonyms) and syntax used for the literature search.

#1: “Antimicrobial Resistance” Terms	#2: “Type of Geographical Setting” Terms	#3: “Environmental” Terms	#4: “Animal Handling or Agriculture” Terms	#5: “Countries in LA” Terms	#6: Combined Search
(“antimicrobial drug resistances” OR “antimicrobial drug resistance” OR “antibiotic resistance” OR “drug resistances, microbial”) OR” antibiotic resistance, microbial” OR “antimicrobial resistance” AND	(rural OR “rural populations” OR “rural settings” OR “rural health services”) AND	(environment * OR water OR soil OR lixiviation) AND	(“animal production” OR animal OR livestock OR agricultur * OR “animal husbandry” OR poultry OR food) AND	(Argentina OR Bolivia OR Brazil OR Chile OR Colombia OR “Costa Rica” OR Cuba OR “Dominican Republic” OR Ecuador OR “El Salvador OR Guatemala OR Haiti OR Honduras OR Mexico OR Nicaragua OR Panama OR Paraguay OR Peru OR Uruguay OR Venezuela)	#1 AND #2 AND #3 AND #4 AND #5
